# Epidemiological data from the COVID-19 outbreak, real-time case information

**DOI:** 10.1038/s41597-020-0448-0

**Published:** 2020-03-24

**Authors:** Bo Xu, Bernardo Gutierrez, Sumiko Mekaru, Kara Sewalk, Lauren Goodwin, Alyssa Loskill, Emily L. Cohn, Yulin Hswen, Sarah C. Hill, Maria M. Cobo, Alexander E. Zarebski, Sabrina Li, Chieh-Hsi Wu, Erin Hulland, Julia D. Morgan, Lin Wang, Katelynn O’Brien, Samuel V. Scarpino, John S. Brownstein, Oliver G. Pybus, David M. Pigott, Moritz U. G. Kraemer

**Affiliations:** 1 Ministry of Education Key Laboratory for Earth System Modeling, Department of Earth System Science, Tsinghua University, Beijing, China; 2 Department of Zoology, University of Oxford, Oxford, United Kingdom; 3 Computational Epidemiology Lab, Boston Children’s Hospital, Boston, United States; 4 Booz Allen Hamilton, Westborough Massachusetts, United States; 5 Mathematical Sciences, University of Southampton, Southampton, United Kingdom; 6 Department of Health Metrics Sciences, University of Washington, Seattle, United States; 7 Mathematical Modelling of Infectious Diseases Unit, Institut Pasteur, UMR2000, CNRS, Paris, France; 8 Network Science Institute, Northeastern University, Boston, United States; 9 Department of Pediatrics, Harvard Medical School, Boston, United States; 10 School of Biological and Environmental Sciences, Universidad San Francisco de Quito USFQ, Quito, Ecuador; 11 School of Public Health, Boston University, Boston, United States; 12 Department of Paediatrics, University of Oxford, Oxford, United Kingdom; 13 School of Geography and the Environment, University of Oxford, Oxford, United Kingdom; 14 Institute for Health Metrics and Evaluation, University of Washington, Seattle, United States; 15 Department of Genetics, University of Cambridge, Cambridge, United Kingdom

**Keywords:** Viral infection, Epidemiology, Viral epidemiology, Risk factors

## Abstract

Cases of a novel coronavirus were first reported in Wuhan, Hubei province, China, in December 2019 and have since spread across the world. Epidemiological studies have indicated human-to-human transmission in China and elsewhere. To aid the analysis and tracking of the COVID-19 epidemic we collected and curated individual-level data from national, provincial, and municipal health reports, as well as additional information from online reports. All data are geo-coded and, where available, include symptoms, key dates (date of onset, admission, and confirmation), and travel history. The generation of detailed, real-time, and robust data for emerging disease outbreaks is important and can help to generate robust evidence that will support and inform public health decision making.

## Abstract (summary)

**Table:** 

Measurement(s)	coronavirus infectious disease • Viral Epidemiology
Technology Type(s)	digital curation
Factor Type(s)	geolocation • date • travel history • age • sex
Sample Characteristic - Organism	Homo sapiens • SARS-CoV-2 • Betacoronavirus

Machine-accessible metadata file describing the reported data: 10.6084/m9.figshare.11974344

## Background & Summary

In December 2019 a number of novel coronavirus-infected pneumonia (NCIP) cases were recorded in a large metropolitan City in China, Wuhan, caused by infection with a novel coronavirus named SARS-CoV-2^1^. The outbreak subsequently spread to other cities in Hubei province and across China. Increasingly, epidemiological studies are performed in real-time during an outbreak to understand key metrics such as the epidemic’s reproduction number, serial interval distribution, incubation period and risk of international spread^2,3^. Geo-positioned records of case data can be important for risk communication and evaluation during outbreaks, especially when they are available in real-time^4,5^.

Epidemiological data is needed during emerging epidemics to best monitor and anticipate spread of infection. In order to provide openly available, accurate and robust data during the COVID-19 outbreak, we collected, and continue to curate, a real-time database of individual-level epidemiological data^6^. Other data sources have been focusing mostly on aggregated case counts per geographic location^7^.

## Methods

We use a range of different sources to update and curate our database. First, we use official government sources and peer-reviewed scientific papers that report primary data as the gold standard for data inclusion. Government sources include press releases on the official websites of Ministries of Health or Provincial Public Health Commissions, as well as updates provided by the official social media accounts of governmental or public health institutions. Second, to find additional details for each case or patient we augment these data with online reports, mainly captured through news websites (e.g., https://www.163.com) or via news aggregators (e.g., https://bnonews.com/). We recorded all data sources in our database. Third, in some instances more detailed data are available, typically through peer-reviewed research articles^1,7^, which were subsequently used to modify existing records in the database. We added a full list of sources that were used to our Github repository (https://github.com/beoutbreakprepared/nCoV2019/blob/master/source_list.csv).

**Fig. 1:**
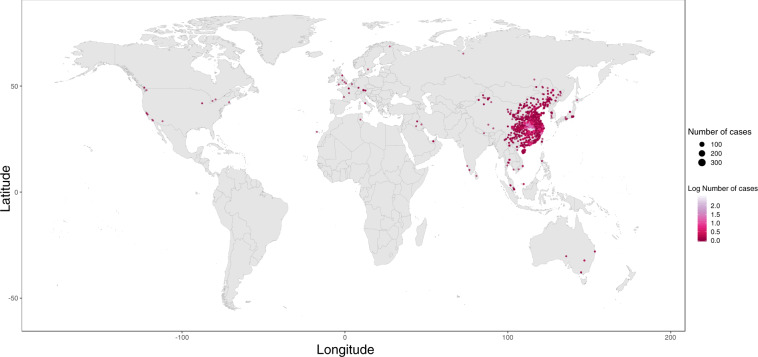
Global distribution of reported confirmed cases from December 1, 2019 to February 5, 2020.

**Fig. 2:**
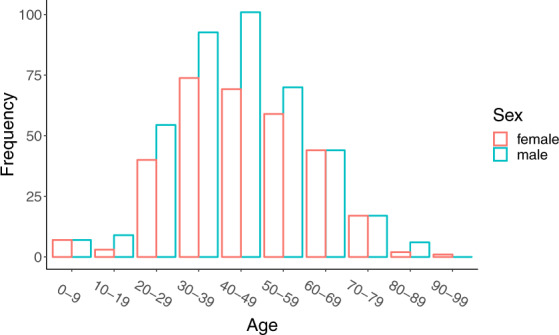
Age and sex distribution of confirmed cases globally (excluding Hubei).

We collected data on the following: (a) Key dates, which include the date of onset of disease, date of admission to hospital, date of confirmation of infection, and dates of travel. (b) Demographic information about the age and sex of patients/cases. (c) Geographic information, at the highest resolution available down to the district level. We excluded information that was at the building level so that cases could not be identified. Geographic information was subdivided into administrative units (admin 0 = country, admin 1 = province, admin 2 = county, admin 3 = city, and where available, specific locations). (d) Symptoms, (e) Any additional information such as exposure to the Huanan seafood market or record of exposure to infected individuals. Summaries of the data are shown in Figs. 1 and 2.

We discussed best-practices among the data curators to reduce the risk of duplicate efforts or erroneous entries. Those include, for example, that some Chinese provinces reported new cases more than once a day, with each report providing only new data. Other provinces provided updates throughout the day and then provided a final update listing all new cases, inclusive of earlier reports. In the latter case, entry of all the newly reported cases would result in duplication of cases from earlier updates. Additionally, as countries began to report asymptomatic PCR-positive individuals, their referencing or indexing of patients sometimes changed. For example, Japan’s Ministry of Health identified novel coronavirus pneumonia cases ordinally up to the country’s eighth case. The next three cases were identified during testing of a Japanese national flown from Wuhan on a charter flight for repatriation. One of those cases became the Ministry of Health’s ninth case while the other two were asymptomatic and not considered the 10th and 11th cases in their press release. As this distinction was not made in other countries, the practice was documented to avoid confusion of cases in the line-list.

## Geo-positioning of Data

Geographical information came in two forms: references to specific settlements, and references to areas, typically administrative units. All geographic metadata was standardized via the use of a common geographic reference table. New unique distinct locations were added to the reference table, and all subsequent entries had geographic information populated from this table. Location names are often duplicated within a country, so contextual information was used to ensure the correct site was selected. When the site name was not found, information from the text was also used to scan sites in the approximate area to check for alternate spellings of the site name. We had curators skilled with the following languages: English, mandarin Chinese, Cantonese, Spanish, and Portuguese. To add new geographies to the database, Google Maps (https://www.maps.google.co.uk) and Google Earth (http://www.google.co.uk/intl/en_uk/earth) were used to determine latitude and longitudes, and relevant administrative metadata was extracted by querying the relevant country reference shapefile. For locations that are administrative units, information was populated by referring to the country reference shapefile, sourced primarily from GADM (https://gadm.org/) with the admin_id field to allow for easier polygon selection.

The distribution of geographic locations where cases have been reported is shown in Fig. 1. To provide real-time visualization we designed an interactive web application using Mapbox and automatically update the results using JavaScript. This visualisation is available at https://www.healthmap.org/ncov2019/.

## Data Records

A static copy of the dataset has been uploaded to figshare^6^, which includes a fixed version of the data record at the time of submission, ranging from 1^st^ December 2019 to 5^th^ January 2020. A live version of the data record, which will be continually updated, can be downloaded from (https://github.com/beoutbreakprepared/nCoV2019) or directly from Google Drive: https://docs.google.com/spreadsheets/d/1itaohdPiAeniCXNlntNztZ_oRvjh0HsGuJXUJWET008/edit#gid=0 in CSV format, that can be imported it into a variety of software programs. We have also established a Github repository available at: https://github.com/beoutbreakprepared/nCoV2019/covid19 and provide code for importing the data into R statistical software. The epidemiological situation regarding the COVID-19 outbreak is continuously evolving. We therefore have made available an archive data folder through our Github repository where new data is uploaded. Each of the rows represents a single individual case and ID. A description of the fields in the database is shown below and is available through a data dictionary on Github (https://github.com/beoutbreakprepared/nCoV2019/covid19):

**ID** - Unique identifier for reported case. Currently ID is run concurrently for cases reported from Hubei, China and cases reported outside of Hubei, China. ID order does not necessarily reflect epidemiological progression, or reporting date, and should not be used to order cases in temporal progression.

**age** - Age of the case reported in years. When not reported, N/A is used. Age ranges are recorded as start_age-end_age e.g. 50–59.

**sex** - Sex of the case. When not reported, N/A is used.

**city** – Initial generic geographic metadata is reported here. Subsequently standardized via lookup with geographic reference table.

**province** – Initial entry of name of the first administrative division in which the case is reported. Subsequently standardized via lookup with geographic reference table.

**country** - Name of country in which the case is reported. Note that imported cases will be assigned to the country in which confirmation occurred - this is typically in the arrival country, rather than the site of infection. “Travel_history_location” will describe other locations of travel for such instances.

**wuhan(0)_not_wuhan(1)** - Binary flag to distinguish cases from Wuhan, Hubei, China, from all other cases. 0 denotes a case is reported in Wuhan, 1 denotes a case reported elsewhere in the world.

**latitude** - The latitude of the specific location (denoted as “point” in “geo_resolution”) where the case was reported, or the latitude of a representative location (denoted as “admin” in “geo_resolution”) within the administrative unit the case is reported.

**longitude** - The longitude of the specific location (denoted as “point” in “geo_resolution”) where the case was reported, or the longitude of a representative location (denoted as “admin” in “geo_resolution”) within the administrative unit the case is reported.

**geo_resolution** - An indicative field in which the spatial representativeness of “latitude” and “longitude” are described. “point” indicates that a specific location is being represented by these coordinates. “admin” denotes that the coordinates are representative of the administrative unit in which coordinates lie. Subsequent “admin3”, “admin2”, “admin1” and corresponding “admin_id” and “shapefile” will allow for a more specific representation to be had.

**date_onset_symptoms** - Date when the reported case was recorded to have become symptomatic. Specific dates are reported as DD.MM.YYYY. Ranges are recorded as DD.MM.YYYY - DD.MM.YYYY. Ranges with uncertain start or finish dates are recorded as - DD.MM.YYYY and DD.MM.YYYY - respectively.

**date_admission_hospital** - Date when the reported case was recorded to have been hospitalized. Specific dates are reported as DD.MM.YYYY. Ranges are recorded as DD.MM.YYYY - DD.MM.YYYY. Ranges with uncertain start or finish dates are recorded as - DD.MM.YYYY and DD.MM.YYYY - respectively.

**date_confirmation** - Date when the reported case was confirmed as having COVID-19 using rt-PCR. Confirmation accuracy is contingent on the data source used. Specific dates are reported as DD.MM.YYYY. Ranges are recorded as DD.MM.YYYY - DD.MM.YYYY. Ranges with uncertain start of finish dates are recorded as - DD.MM.YYYY and DD.MM.YYYY - respectively.

**symptoms** - List of symptoms recorded in the description of the case.

**lives_in_Wuhan** - Recorded relationship of patient with city of Wuhan, Hubei, China. “yes” indicates that the case was a resident of Wuhan. “no” indicates that the case is not a resident of Wuhan (residential). No information indicates that no data was available.

**travel_history_dates** - Recorded travel dates to and from Wuhan. Specific dates are reported as DD.MM.YYYY and indicate date when the individual left Wuhan. Ranges are recorded as DD.MM.YYYY - DD.MM.YYYY when both are available. Ranges with uncertain start of finish dates are recorded as - DD.MM.YYYY and DD.MM.YYYY - respectively.

**travel_history_location** - An open field describing the recent recorded travel history of the case.

**reported_market_exposure** - An open field indicating “yes” if there was reported market exposure and “no” if there was not. N/A indicates that no information is provided.

**additional_information** - Any additional information that may be informative about the case, such as the occupation of the patient, the purpose of their travels, the hospital they were admitted to, etc.

**chronic_disease_binary** - 0 represents a case that was reported to have no chronic disease and 1 represents cases that reported a chronic disease

**chronic_disease** - Reported chronic condition(s) of the reported case.

**source** - URL identifying the source of this information

**sequence_available** - If there was a genomic sequence available the accession number is inserted here.

**outcome** - Patients outcome, as either “died” or “discharged” from hospital.

**date_death_or_discharge** - Reported date of death or discharge in DD.MM.YYYY format.

**location** – Location of the reported case.

**admin3** – Administrative unit level 3 (e.g., zip code) of where the case was reported.

**admin2** – Administrative unit level 2 (e.g., county) of where the case was reported.

**admin1** – Administrative unit level 1 (e.g., province) of where the case was reported.

**country_new** – Administrative unit level 0 (e.g., country) of where the case was reported.

**admin_id** – Administrative unit ID of the lowest level available for the case reported.

At time of publication the database contained 18,529 geopositioned records from December 1, 2019 to February 5^th^, 2020 (Fig. 1). A map of all records can be viewed in real-time here: https://www.healthmap.org/ncov2019/.

Reference shapefiles are available via ESRI (https://esri.maps.arcgis.com/home/item.html?id=c9c26d32bdec4beea7589e303bb06a85 for China admin1, https://esri.maps.arcgis.com/home/item.html?id=0a57592fd41344649f59738e5c330fd3, for China admin2 https://ihme.maps.arcgis.com/home/item.html?id=f3517e223cd544e5a80e9d142caae2b4 for China admin3, https://esri.maps.arcgis.com/home/item.html?id=c8c9696ee6454fb297e36b7dac91481c for Hong Kong, and https://esri.maps.arcgis.com/home/item.html?id=6f76647cf3804e24bd205eb21fccdbc4 for Macau), and GADM (https://gadm.org/data.html for rest of world). All shapefiles have a unique identifier for each component – admin_id should be used to merge the line list data with the relevant shapefiles for a given country, and administrative tier. The admin_id for points refers to the lowest tier of administrative unit reported in columns admin3, admin2, admin1, and country_new. For administrative units themselves, the relevant administrative layer to use is denoted by the geo_resolution column.

## Technical Validation

After initial data entry the database was checked using two complementary methodologies to identify possible duplicate records. One was a machine enabled one and the other was done manually by the data curators. The first algorithm proceeds in 5 steps. (1) columns with no variability across all records were removed, (2) the remaining data were hashed using a 32-bit variant of MurmurHash3 implemented in the R package *FeatureHashing* version 0.9.1.3^8,9^, (3) a principle component analysis on the centered, scaled hashed feature matrix is performed for dimension reduction, with principle components having standard deviations greater than 0.5 retained, (4) pairwise, Euclidean distances are then calculated and are normalized based on the smallest observed distance between records, and (5) records that have pairwise distances less than the 0.5th percentile are flagged as duplicates. Duplicate are defined as cases that refer to the same case. Code for these methods is hosted on our GitHub repository (https://github.com/beoutbreakprepared/nCoV2019/covid19). Records identified as possible duplicates were communicated to data curators via Github and flagged in the database. Curators then discussed amongst themselves via an online chat system (www.slack.com) to reach a consensus on how to address the possible duplications.

## Usage Notes

These data can be used to investigate the epidemiological COVID-19 outbreak in China and elsewhere. This includes descriptive mapping of occurrences through time and estimation of key epidemiological parameters using mathematical models. The data are openly available and we will continue to curate the database as new information is made available. However, if the epidemic continues to grow then public health agencies are unlikely to continue to report individual-level case data, and instead will switch to reporting only total numbers (or estimates thereof) of confirmed or suspected cases as done for previous large outbreaks such as pandemic flu H1N1^10^. When detailed data becomes increasingly less available as the epidemic grows we may transition to an augmented database structure that only reports total new cases per location. Other groups have presented similar datasets which are complimentary to the one presented in this publication^11^. However, the dataset presented here includes fine grained geographic details and the most comprehensive list of cases.

While every effort has been made to standardize the geographic representation of cases, with a common source of reference shapefiles outlined, when considering geographic analysis of the data a few limitations must be acknowledged. The first is that, while native speakers were consulted wherever possible, there is the possibility of transliteration errors occurring when extracting data from native language into English-script analogues. This is most likely to happen when looking at point data. We have provided sources consulted so that users may cross-reference the original source wherever this is may be an issue. While administrative units are supplied with shapefiles sources and unique identifiers with these files so that users can understand the corresponding geographic scale which the row represents, with points, different settlements cover different spatial extents. Should users wish to incorporate this information into spatially-dependent models, they should exercise caution in possible misrepresentation of geographic specificity. We recommend that sensitivity of results could be evaluated by using buffers around point latitude and longitudes, or cross-referencing city-gazettes.

There are possible changes of reporting during the first month of the outbreak. For example, we find that demographic information reported initially as case numbers were small but detailed case information became less available after the 23^rd^ of January. Initial cases from Wuhan are well described, mostly thanks to epidemiological studies published towards the end of January^7^. Even though we made the best attempt to report data as accurately as possible, given the dynamic nature of the outbreak we caution that the database cannot be guaranteed to be free from error, and we apologize in advance if there are missing entries that were not picked up using our standardised protocol^12^. Going forward we will likely update records in the period described here which occurs frequently after outbreaks^13–16^. We encourage users of the database to contact us directly if potential errors or omissions have been found. This can be done by either emailing the corresponding authors or, preferably, by submitting a request via the Github repository (https://github.com/beoutbreakprepared/nCoV2019).

## Data Availability

All code used to clean data has been uploaded to the repository and is also on our Github page: https://github.com/beoutbreakprepared/nCoV2019/tree/master/covid19/src.
